# Piston Error Automatic Correction for Segmented Mirrors via Deep Reinforcement Learning

**DOI:** 10.3390/s24134236

**Published:** 2024-06-29

**Authors:** Dequan Li, Dong Wang, Dejie Yan

**Affiliations:** Space Optics Department, Changchun Institute of Optics, Fine Mechanics and Physics, Chinese Academy of Sciences, Changchun 130033, China

**Keywords:** segmented mirrors, deep reinforcement learning, co-phase error

## Abstract

The segmented mirror co-phase error identification technique based on supervised learning methods has the advantages of simple application conditions, no dependence on custom sensors, a fast calculation speed, and low computing power requirements compared with other methods. However, it is often difficult to obtain a high accuracy in practical application situations with this method because of the difference between the training model and the actual model. The reinforcement learning algorithm does not need to model the real system when operating the system. However, it still retains the advantages of supervised learning. Thus, in this paper, we placed a mask on the pupil plane of the segmented telescope optical system. Moreover, based on the wide spectrum, point spread function, and modulation transfer function of the optical system and deep reinforcement learning—without modeling the optical system—a large-range and high-precision piston error automatic co-phase method with multiple-submirror parallelization was proposed. Finally, we carried out relevant simulation experiments, and the results indicate that the method is effective.

## 1. Introduction

In order to pursue a higher imaging resolution, both space-based and ground-based telescopes are being developed in the direction of a larger aperture. However, the larger the size of the telescope’s primary mirror, the more difficult to design, process, manufacture, and verify it is. The emergence of segmented mirrors has greatly reduced the quality of primary mirrors, processing costs, manufacturing cycles, and the difficulty of transportation and launch. Based on the position of the reference submirror, other submirrors of the segmented primary mirror have six degrees of freedom: piston aberration along the optical axis (z-axis), tip-tilt along the x and y axes, movement in the x and y planes, and rotation around the optical axis. However, the first three of these factors have the biggest impact on image quality, and they are collectively known as the co-phase error. In order to bring the overall imaging quality of the segmented telescope close to the level of the diffraction limit, it is required that the submirrors have an extremely high co-phase accuracy [1,2].

At present, an accurate co-phase of the submirrors mainly includes two steps of submirror co-phase error detection and submirror error correction, where submirror error correction can be achieved with a multi-dimensional precision displacement platform. According to this principle, successfully applied co-phase error detection methods can be divided into pupil surface detection methods based on specific hardware sensors [3,4,5,6] and methods based on focal plane image information [7,8,9,10,11,12,13].

Among these methods, methods based on deep learning have been rapidly developed and widely studied in recent years because they only rely on focal plane image information, have a fast processing speed, can extend the detection range through multi-wavelength or wide spectra, and can be applied to point targets and extended targets at the same time. However, in these methods, in order to achieve a high submirror co-phase accuracy, a high registration accuracy is required between the simulation model of the optical system used to generate the training dataset and the actual optical system, if the neural network training dataset is generated by the simulation model. However, this problem is difficult to solve; it is very difficult to obtain an accurate training dataset using real system data. The problem has greatly hindered the practical application of this method.

The deep reinforcement learning algorithm [14] is composed of deep learning and reinforcement learning, in which deep learning is responsible for the low-dimensional feature extraction of high-dimensional data, like our eyes, and reinforcement learning uses the Markov process to make judgments and decisions, like our brain. This approach combines the higher perceptual capabilities of deep learning and the decision-making capabilities of reinforcement learning. Through a continuous interaction with the external environment, deep reinforcement learning algorithms can learn how to perform correct operations under complex conditions without modeling the external environment in the process. Therefore, in this paper, we placed a mask on the pupil plane of the segmented telescope optical system. Additionally, based on the wide spectrum, point spread function (PSF), and modulation transfer function (MTF) of the optical system and deep reinforcement learning, we implemented an automatic co-phase method for the multiple-submirror piston errors of segmented telescopes. This method does not need to model segmented telescopes and retains the advantages of not requiring iterations and does not depend on custom sensors. It also has the low computing power requirements of the deep learning co-phase error correction method. In reference [15], the author used the reinforcement learning method to correct the piston error of two submirrors in a three-submirror optical system. Compared with the methods in reference [15], the optical system used in this method is more complex and the correction efficiency is higher.

## 2. Method

### 2.1. Deep Reinforcement Learning

In recent years, with the development of deep learning technology, deep reinforcement learning algorithms have been more widely used, the most famous of which is the AlphaGo system [16] developed by the DeepMind (USA) team. With this algorithm as the core, it overwhelmingly defeated the Go World Champion Lee Sedol.

Reinforcement learning [14] is an integral part of machine learning. Its biggest feature is learning through interaction. Its goal is to train an agent to output a series of decisions based on the feedback of the environment, instead of only one result, which is similar to the human brain.

The reinforcement learning algorithm is mainly composed of an agent, an environment, value function ν, action policy function π, and reward function R. The agent maximizes the cumulative reward by sensing the state of the environment and learning to choose a series of actions according to the reward function value of the environment’s feedback.

The action policy function π defines the behavior of the agent in a given state of the external environment and maps from a state to a behavior, it is the core of the reinforcement learning system.

The reward function R defines the learning goal of reinforcement learning. In each time step, after the agent makes a corresponding action according to the environment state and action policy function π, the reward function will output the corresponding reward value to the agent, which defines the performance of the agent in the environment.

The concept of a long-term reward is commonly used in reinforcement learning and is defined by Equation (1), where γ is the discount factor and $R_{t}$ is the reward function value obtained at the time step *t* of the agent.

(1) $$G_{t}=R_{t}+\gamma \cdot R_{t+1}+\gamma^{2}\cdot R_{t+2}+\gamma^{3}\cdot R_{t+3}\ldots \ldots$$

The value function ν represents the value of the environmental state of the agent. More specifically, the state value function describes the reward function expectations of the agent in a given state based on a particular policy function π. In addition, an action value function can also be defined. The value function is crucial to reinforcement learning, as it allows the agent to determine the quality of the state of its current environment and the quality of the action policy π.

The environmental model is the simulation of environmental state transfer. When the external environment state and the output behavior of the agent are given, we can obtain the environmental state of the next moment according to the environmental model. In this paper, the environment is the segmented optical system. Reinforcement learning does not always require the environment model, it can be divided into two types: model-based and model-free. Model-free reinforcement learning algorithms mainly learn through analysis of the policy function and the value function. The proximal policy optimization algorithm subsequently used in this paper is a model-free method, so there is no need to model the segmented mirror optical system.

**Figure 1:**
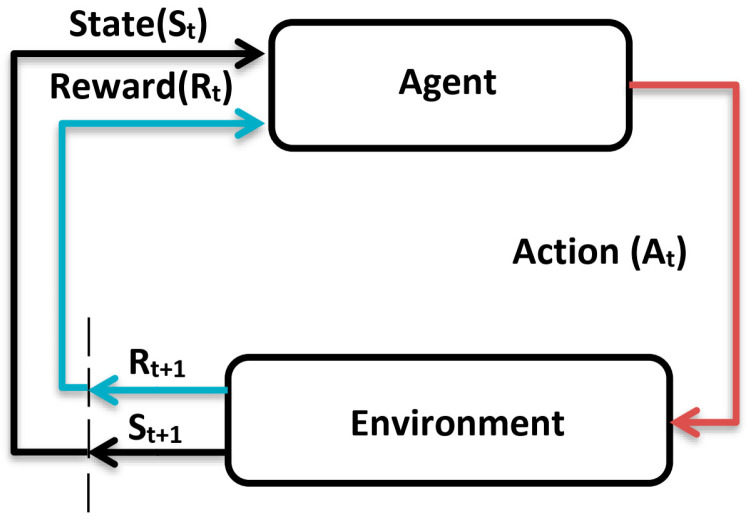
The schematic diagram of the reinforcement learning algorithm.

On the whole, in reinforcement learning, the agent chooses corresponding actions to perform in different environmental states according to the policy function π, the environment feedbacks the new state and reward value according to the actions of the agent, the agent chooses new actions according to the new state, and the cycle continues until the end of the training. This process is called an episode. In deep reinforcement learning, both the policy function π and the value function ν are composed of neural networks, so we hope to maximize the cumulative reward obtained by an episode by adjusting the parameters of policy function and value function; thus, the reinforcement learning algorithm will ultimately translate into the optimization problem of how to design an agent that maximizes long-term reward in the environment. The reward function is only used to update the parameters of the agent during the training process and is no longer needed after the training is completed. The schematic diagram of the reinforcement learning algorithm is shown in Figure 1.

### 2.2. Proximal Policy Optimization (PPO)

Proximal policy optimization (PPO) [17], a reinforcement learning algorithm proposed by OpenAI in 2017, is considered to be the current state-of-the-art method in the field of reinforcement learning, this method is used in the recently popular ChatGPT [18]. It is a method of an on-policy, online, model-free, policy gradient which used to solve continuous or discrete action spaces. The piston error correction of the segmented mirror in this paper belongs to the continuous action space, so this paper will build the corresponding method based on the PPO algorithm. Compared with other algorithms, PPO’s strategy update is more stable, so the correction operation of submirrors is safer during training. This method has a good scalability for large-scale problems, and it can be used to deal with complex tasks by increasing the scale of the network and using distributed computing methods.

In PPO, the policy is represented by actor policy network π(A|S;θ), the input of which is the observation quantity of the environment state of the agent. In this paper, the output of the actor network is the piston error correction quantity of the segmented telescope, so it belongs to the continuous action space problem. Therefore, the output of the actor network in the PPO algorithm used in this paper is the mean and standard deviation of the Gaussian probability distribution of taking each continuous action A when in state S of the environment. The value function consists of the critic network V(S;ϕ), whose input is the observation quantity of the environment state in which the agent is located, and whose output is the long-term discount reward R that the agent can achieve under the current actor policy network π(A|S;θ).

The training process of PPO is as follows:

1. Randomly initialize the parameter ϕ of the critic network and the parameter θ of the actor network;

2. Use the current actor policy network π(A|S;θ) to make the agent interact with the environment many times, so as to obtain multiple pairs of data composed of the environmental state, the action, and the corresponding reward values:

$$S_{ts},A_{ts},R_{ts+1},S_{ts+1},A_{ts+1},R_{ts+2}\ldots \ldots \ldots \ldots S_{ts+N-1},A_{ts+N-1},R_{ts+N},S_{ts+N}.$$

where $S_{t}$ is the state observation quantity of the environment at time *t*, $A_{t}$ is the corresponding action taken by the agent according to the observed environmental state $S_{t}$, the actor policy network π(A|S;θ), and the output action probability distribution. $S_{t+1}$ is the observation quantity of the environmental state at the next moment.$R_{t+1}$ is the value of the reward function obtained by the environment moving from $S_{t}$ to $S_{t+1}$, and t is the starting time step of the current set of N experiences.

3. For each episode step *t* = *ts* + 1, *ts* + 2, …, *ts* + *N*, compute the advantage function $D_{t}$ by generalized advantage estimator [19], which is the discounted sum of temporal difference errors.

(2) $$D_{t}={\sum_{K=t}^{ts+N-1}\left(\gamma \beta \right)}^{k-t}\delta (R_{t}+b\gamma V(S_{t};\phi ))$$

Here, b=1, if $S_{ts+N}$ is not a terminal state and b=0 otherwise. β is a smoothing factor. γ is the discount factor. Compute the return $G_{t}$.

(3) $$G_{t}=D_{t}+V(S_{t};\phi )$$

4. Sample a random mini-batch dataset of size Q from the current set of experiences. Each element of the mini-batch dataset contains a current experience and the corresponding return and advantage function values.

Update the parameters of the critic net by minimizing the loss $L_{critic}$ and across all sampled mini-batch data.

(4) $$L_{critic}(\phi )=\frac{1}{2Q}{\sum_{i=1}^{Q}\left(G_{i}-V(S_{i};\phi )\right)}^{2}$$

Update the parameters of the actor policy net by minimizing the actor loss function $L_{actor}$ across all sampled mini-batch data,

(5) $$L_{actor}(\theta )=\frac{1}{Q}\sum_{i=1}^{Q}\left\{-min\left[r_{i}(\theta )\cdot D_{i},c_{i}(\theta )\cdot D_{i}\right]+wH_{i}(\theta ,S_{i})\right\}$$

where $r_{i}(\theta )=\pi (A_{i}|S_{i};\theta )/\pi (A_{i}|S_{i};\theta_{old})$, $c_{i}(\theta )=max\left\{min\left[r_{i}(\theta ),1+\varepsilon \right]),1-\varepsilon )\right\}$, $D_{i}$ is the advantage function for the i-th element of the mini-batch, and $G_{i}$ is the corresponding return value.

$\pi (A_{i}|S_{i};\theta )$ and $\pi (A_{i}|S_{i};\theta_{old})$ are the probability of taking action $A_{i}$ when in state $S_{i}$, given the updated policy parameter θ and given the previous policy parameter $\theta_{old}$ from before the current learning epoch, respectively. ε is the clip factor. $H_{i}(\theta )$ is the entropy loss. ω is the entropy loss weight factor, which promotes the agent to exploration.

5. Repeat Steps 2 through 4 until the training episode reaches a terminal state.

### 2.3. Piston Error Automatic Correction Technology for Optical Segmented Mirrors via PPO

In the process of the co-phase adjustment of segmented telescopes, the co-phase errors of submirrors are divided into piston errors (along the optical axis) and tip/tilt errors (which rotate around the x/y axis). The research of reference [20] shows that tip/tilt errors have a lower impact than piston errors in the imaging of segmented primary mirrors. In this paper, we will use the PPO to implement the automatic correction of the piston error of the segmented mirror. Theoretically, this method does not require the optical model and only needs the constructed reward function value and focal plane image. Thus, the method avoids the problem that the simulation model has difficulty in accurately registering with the actual model in the methods based on supervised learning [21,22,23]. In addition, since the algorithm maximizes the value of the reward function obtained by constantly adjusting the position of the submirror, the absolute positioning accuracy of the submirror high-precision adjustment platform is less required, and only its resolution is required; this advantage further reduces the difficulty of the method implementation.

For the deep reinforcement learning algorithm, the reward function is very important: it should effectively represent the piston error of each submirror. We construct the reward function using the method in references [24,25,26,27], which is briefly explained below.

**Figure 2:**
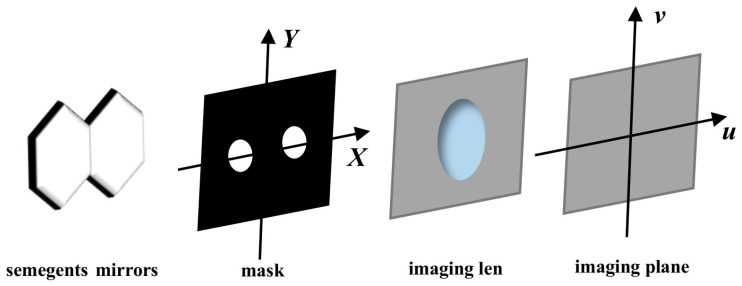
The schematic diagram of mask and segmented mirrors.

As can be seen in Figure 2, a double-hole mask is placed on the exit pupil surface of the segmented mirror imaging system, which can pass through the reflected light of the two submirrors. Assuming that the incident light is a wide spectrum, $\lambda_{0}$ is the central wavelength, and Δλ is the bandwidth of the wide spectrum, the PSF expression of the optical system can be expressed by Equation (6).

(6) $$PSF_{b}(x,y,\lambda )=2\int_{\lambda_{0}-\frac{\Delta \lambda }{2}}^{\lambda_{0}+\frac{\Delta \lambda }{2}}PSF_{m}(x,y,\lambda )d\lambda$$

where $PSF_{m}(x,y,\lambda )$ is the point spread function in the case of monochromatic light, and its expression is as follows:

(7) $$PSF_{m}(x,y,\lambda )=2\frac{\frac{D^{2}}{2}J_{1}^{2}(\pi D\sqrt{{\left(\frac{x}{\lambda f}\right)}^{2}+{\left(\frac{y}{\lambda f}\right)}^{2}})}{\sqrt{{\left(\frac{x}{\lambda f}\right)}^{2}+{\left(\frac{y}{\lambda f}\right)}^{2}}}\times [1+cos\frac{2\pi }{\lambda }(p-\frac{B}{f}x)]$$

where p is the piston error, B is the center distance between two subpupils, D is the diameter of the subpupil, $J_{1}(\cdot )$ is the first-order Bessel function, and f is the focal length of the imaging lens. Equation (7) can be approximately expressed as a differential summation, the range is divided into *n* intervals equally, and the equation is as follows:

(8) $$PSF_{b}(x,y,\lambda )=\sum_{i=1}^{n}\left[\frac{PSF_{m}(x,y,\lambda_{i})\Delta \lambda }{n}\right]=\frac{\Delta \lambda }{n}\sum_{i=1}^{n}\left\{\begin{array}{l} 2\frac{\left(\frac{D^{2}}{2})f^{2}\lambda_{i}^{2}J_{1}^{2}(\frac{\pi D}{\lambda_{i}f}\sqrt{x^{2}+y^{2}}\right)}{x^{2}+y^{2}}\times \\ \left[1+cos\frac{2\pi }{\lambda_{i}}(p-\frac{B}{f}x)\right] \end{array}\right\}$$

The MTF can be given by Equation (9), where $MTF_{sub}$ is the MTF of a single aperture diffraction limited imaging system in which the aperture diameter is D, $f_{x}=x_{0}/\lambda f$, $f_{y}=y_{0}/\lambda f$. If ρ≥D/λf, $MTF_{sub}=0$, else $MTF_{sub}$ is shown in Equation (10).

(9) $$\begin{array}{l} MTF(f_{x},f_{y})=\left|FT\right.\left.\left[PSF_{b}(x,y,\lambda )\right]\right|=2MTF_{sub}(f_{x}+f_{y})+\\ \left[MTF_{sub}(f_{x}+B/\lambda f,f_{y})+MTF_{sub}(f_{x}-B/\lambda f,f_{y})\right] \end{array}$$

(10) $$MTF_{sub}=\frac{2}{\pi }(arccos(\frac{\lambda f}{D}\rho )-(\frac{\lambda f}{D}\rho )\sqrt{1-{\left(\frac{\lambda f}{D}\rho \right)}^{2}})$$

According to Equation (9), the MTF is composed of two side-lobes and one center peak. The center normalized amplitude of the MTF side-lobe ($MTF_{nph}$) decreases with the increase of the piston, when the value of the piston reaches half the coherence length, $MTF_{nph}$ will be zero. The coherence length L can be given by Equation (11), at the same time, this method can break ambiguity of 2π and extend the capture range to half of the coherent length L. The relationship between the piston and the $MTF_{nph}$ can be given by Equation (12).

(11) $$L=\lambda_{0}^{2}/\Delta \lambda$$

(12) $$MTF_{nph}=\frac{1}{n}\sqrt{n+2[\sum_{j=1}^{n-1}\sum_{i=j+1}^{n}cos(\frac{2\pi }{\lambda_{j}}p-\frac{2\pi }{\lambda_{i+1}}p)]}$$

**Figure 3:**
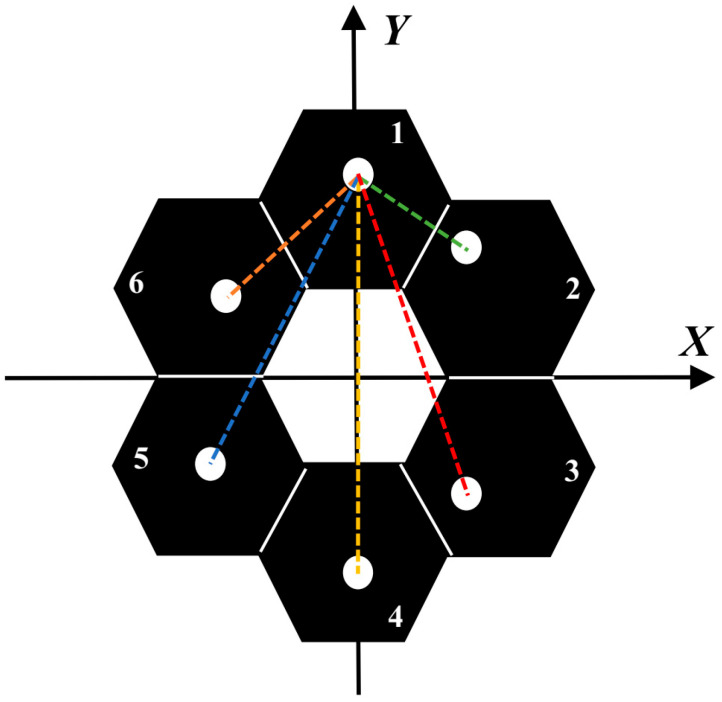
The schematic diagram of the segmented mirror structure and the configuration of the 6 subpupils mask.

If a multihole mask is placed in a multi-submirror segmented optical system and the position of Submirror 1 is taken as the reference, as long as there is no overlap under each interference baseline, that is, the MTF of the optical system is a non-redundant MTF, Equation (12) is still true. Therefore, the amplitude of each $MTF_{nph}$ can effectively represent the piston error of each submirror. Therefore, in this paper, we place the multihole mask at the corresponding position to obtain the $MTF_{nph}$ of each submirror and reference submirror, and sum the amplitude of each $MTF_{nph}$ as the reward function of the reinforcement learning algorithm. We designed the corresponding mask according to the method in reference [25]. The shape of the mask is set up as shown in Figure 3, where the position of Submirror 1 is used as the reference, and the dotted lines of each color are the interference baseline of other numbered submirrors and Submirror 1.

**Figure 4:**
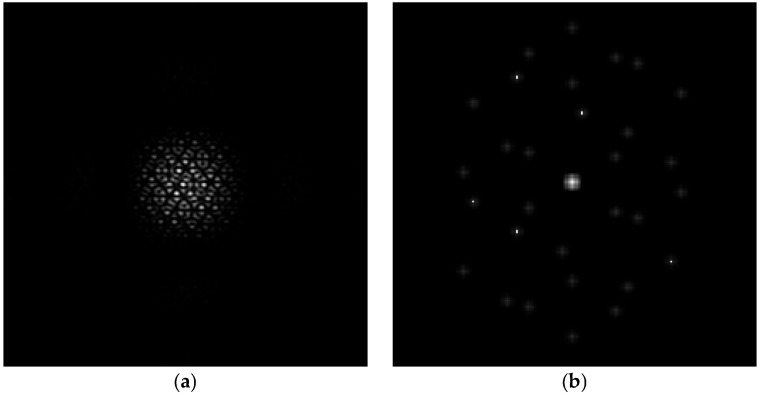
The PSF (**a**) and the MTF (**b**) of the segmented mirror with the mask.

The PSF and MTF of the corresponding optical system are shown in Figure 4a,b. The yellow star points in Figure 4b are the $MTF_{nph}$ collected to reflect the piston errors of each submirror. In reference [28], the author proved that the focal plane image in the segmented telescope optical system containing a multihole mask system has a good ability to represent the piston error of the submirrors. Therefore, in this paper, we take the focal plane image as the observation quantity of the external environment.

**Figure 5:**
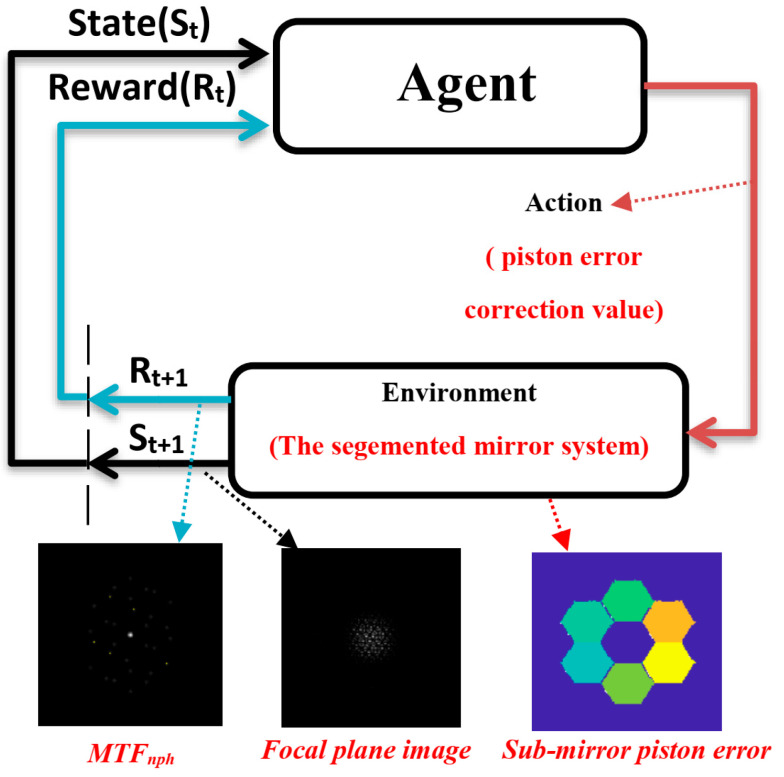
The schematic diagram of the reinforcement learning algorithm in the paper.

To sum up, in this paper, we implement an automatic piston error correction method of the segmented mirror based on the PPO, as shown in Figure 5 below, in which the focal plane image of the optical system after adding a multihole mask will be used as the environmental observation quantity, the $MTF_{nph}$ sum of each submirror and the reference submirror will be used as the reward function R, and the segmented telescope will be used as the interaction environment with the agent. The agent will directly output the piston error correction quantity of each submirror according to the input focal plane image of the segmented mirror optical system, and it will output the next correction quantity of each submirror according to the re-input focal plane image after the submirror’s correction. In this process, the agent will update the critic network V(S;ϕ) and actor policy network π(A|S;θ) according to the reward function, and this process will continue to cycle until the end of training. A well-trained agent will learn an actor policy network π(A|S;θ) that can precisely correct each submirror piston error under any submirror piston error condition.

## 3. Simulation

**Figure 6:**
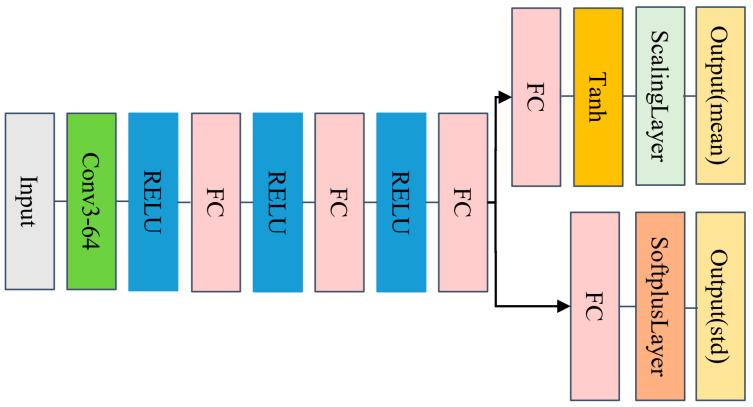
The structure diagram of the critic network.

**Figure 7:**
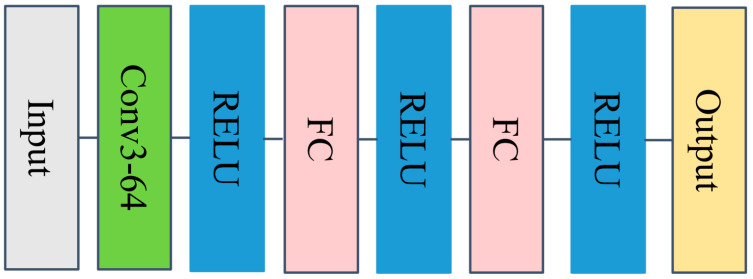
The structure diagram of the actor policy network.

In the PPO algorithm, we need to build the critic network V(S;ϕ) and actor policy network π(A|S;θ). The network structures of both are shown in Figure 6 and Figure 7, respectively. The motivation for adopting a simple architecture is that it is easy to use when prototyping new problems, thereby eliminating the need for simultaneous architectural exploration and hyperparameter optimization. In future research work, we will use more complex network structures to explore whether the corresponding experimental effects can be improved.

The central wavelength of the spectrum is 632.8 nm and the bandwidth Δλ=63.28 nm, so the coherence length L=6328 nm can be calculated according to Equation (11). Therefore, the piston error range of the submirror in this paper is set to [−5λ,5λ]. We use MATLAB to model the segmented telescope, which contains 6 hexagonal submirrors, the pixel size of the imaging detector is 2.5 µm, the F# is 10, and the size of the exit pupil plane is 256 pixels. The mask is set according to the method mentioned in the reference [24,25,26]. The diameter of the mask hole is 8 pixels. The outer circle of the submirror is 40 pixels in diameter.

In order to effectively improve the exploration efficiency in state space and reduce the training difficulty, in contrast to the reference [15], multi-step reinforcement learning is adopted in this paper. The maximum number of steps of each episode is 30 and the discount factor γ=0.97. Therefore, this method can also be used as a long-term maintenance method for the position of the submirrors.

The training learning rate is set to 0.0001, the maximum number of episodes is set to 100,000, and the cumulative reward value of each episode will be recorded; if the average of the cumulative reward value of the 20 most recently trained episodes exceeds 29, the training will stop and the agent will be saved.

In the process of algorithm training, we separately add up the piston error correction amount of each submirror output by the actor policy network after each episode starts, so as to judge whether the piston error of each submirror after correction is still within the correction range. When this accumulation amount is beyond the correction range boundary, even if episode does not reach the maximum number of steps this time, the episode will also end in advance and be judged as an error correction failure (Done = 1), and the reward function will be −1 (this method can still be used for the actual segmented telescope, without affecting the authenticity of the simulation system); otherwise, the reward function will be the sum of each normalized amplitude of $MTF_{nph}$.

**Figure 8:**
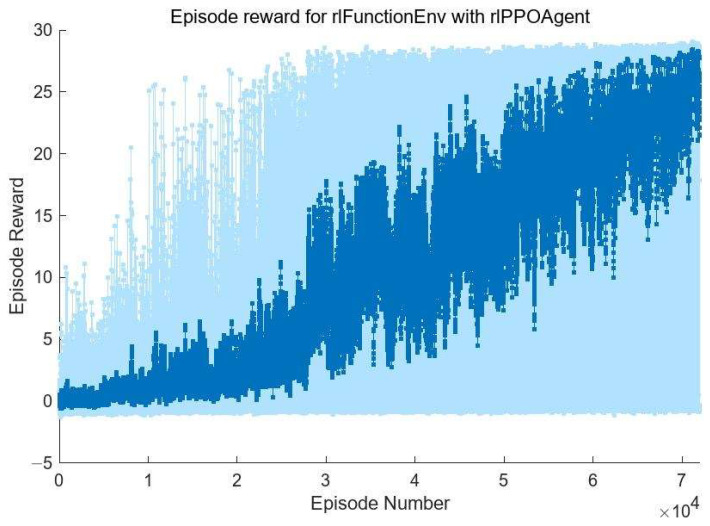
The diagram of the change in the reward function obtained by the agent with the number of episodes.

The training process is shown in Figure 8. The horizontal axis is the number of episodes, and the vertical axis is the cumulative reward function value obtained by the agent in each episode. As can be seen from Figure 8, in the initial training of the agent, due to the poor output of the action strategy network, the piston errors of each submirror often exceed the correction range after several corrections, so the episode is forced to terminate even though the maximum number of steps is not reached. In this case, the value of the reward function will be assigned as −1; that is, the agent will be punished accordingly. In this case, the agent will update the value network and the action strategy network to reduce the probability of this situation occurring in the subsequent training process. With the progress of training, the agent has gradually begun to learn how to automatically correct the piston errors of each submirror. In the later stage of training, the situation in which the piston errors of submirror are outside the correction range due to the error of correction quantity output is basically eliminated. Meanwhile, the cumulative reward value of each episode is also increasing. This means that in the process of correcting 30 times each episode, each submirror piston error is corrected faster and more accurately. However, in order to improve the efficiency of the agent’s exploration of the environment, entropy loss weights promote agent exploration by imposing penalties for being too certain of the actions to take (doing so helps to agents move away from local optima), so there are large fluctuations in the value of the reward function in Figure 8.

**Figure 9:**
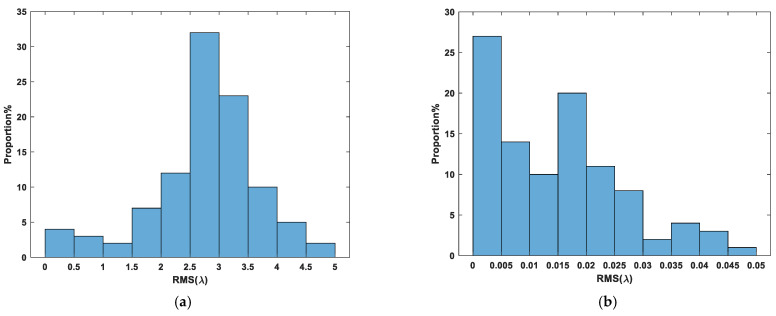
The histogram of segmented mirror piston errors before (**a**) and after (**b**) correction by the deep reinforcement learning algorithm (100 groups experiments).

We saved the trained deep reinforcement learning network model and used the model to correct the randomly initialized 100 groups’ submirror piston errors in the range of [−5λ,5λ] and recorded the relevant experimental results, as shown in Figure 9. It can be seen from Figure 9 that the maximum RMS of the corrected co-phase error is less than 0.05λ, the average RMS is 0.0146λ, and the RMS value is less than 0.025λ for 81 out of 100 corrections, among which the minimum value is 0.0001889λ. Thus, all of the segmented mirror systems which were corrected are known to have a Strehl ratio greater than 0.8 [29].

In order to further reduce the implementation difficulty of this method in the actual system, reducing the number of the training times and applying the method to a system with more submirrors, we adopt the multi-agent reinforcement learning [30] method to train five agents at the same time, and each agent carries out the piston error correction for each submirror, respectively. Although this method seems to require more network parameters to be trained, because each single agent is trained separately, the training difficulty is much lower.

**Figure 10:**
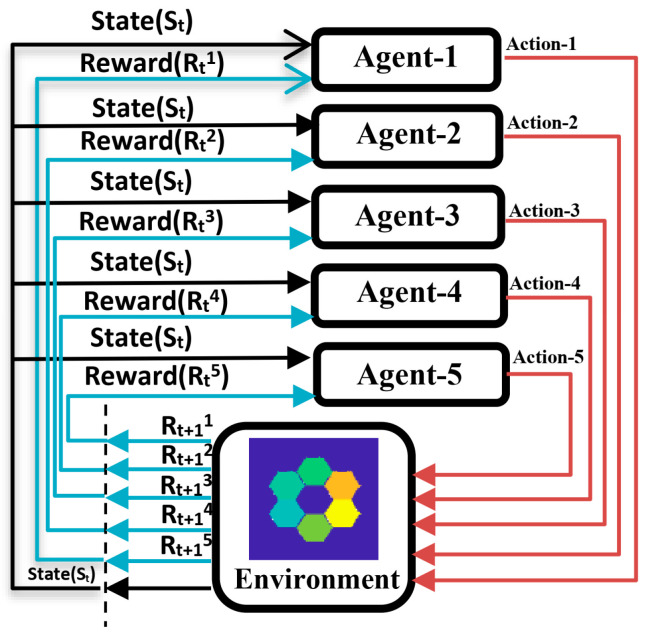
The schematic diagram of automatic correction of submirrors piston error based on multi-agent deep reinforcement learning.

It can be seen from the previous text that the $MTF_{nph}$ amplitude of each submirror and reference mirror is inversely proportional to its own piston error, and it has nothing to do with the piston error of other submirrors. Therefore, in the multi-agent reinforcement learning algorithm in this paper, the reward function of each agent is constructed by the $MTF_{nph}$ of each submirror itself. Each agent has the same environmental observation which is the focal plane image of the optical system. The structure of the action strategy network and value network of each agent is the same as in the previous section. The output of the action policy network of each agent is the quantity of piston error correction for each submirror, and the output of each action policy network is only one dimension. The schematic diagram of the automatic correction of submirrors’ piston error based on multi-agents is shown in Figure 10.

**Figure 11:**
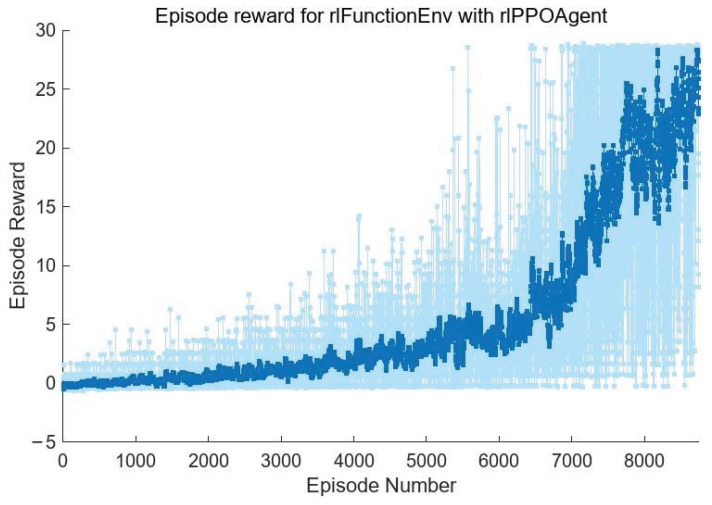
The diagram of the change in the reward function obtained by Agent 2 with the number of episodes.

**Figure 12:**
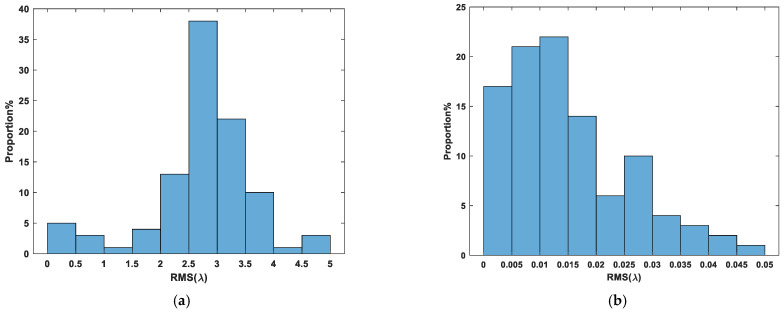
The histogram of segmented mirror piston error before (**a**) and after (**b**) correction by multi-agent deep reinforcement learning (100 groups experiments).

Here, we only show the training process of the agent used to correct submirror 2, as shown in Figure 11 below. The training process of other agents is similar. As can be seen from Figure 11, the number of training is significantly reduced. As before, we also saved multi-agent reinforcement learning and used the models to correct the randomly initialized 100 groups submirror piston errors in the range of [−5λ,5λ] and recorded the relevant experimental results, as shown in Figure 12. It can be seen that the maximum RMS of the corrected co-phase error is less than 0.05λ, the average RMS is 0.0152λ, and the RMS value is less than 0.025λ for 80 out of 100 corrections, among which the minimum value is 0.0002691λ. Therefore, the results of the two methods are similar, but in the multi-agent reinforcement learning training process, the interaction between the agent and the environment is less, and the training difficulty is lower.

Of course, we can also not use the multi-agent method but simply train five agents in turn; this method is also effective. But in this multi-agent method, during each experience in the training process, the five sub-agents interact with the agent by correcting the corresponding submirror, these experiences will be collected, and the parameters of the five sub-networks will be updated simultaneously. Thus, the interaction times between the agent and the environment is reduced, and the sample utilization and training efficiency are improved.

## 4. Conclusions

In this paper, we propose a large-range and high-precision segmented mirror piston error automatic correction method based on deep reinforcement learning. This method does not need to model the segmented mirror system in the training process, so as to avoid the problem of difficult registration between the simulation model and the actual model in the supervised learning method and to be more in line with the application scenario. At the same time, because this method is based on wide spectral imaging, the piston error correction range can be extended by 10 wavelengths (theoretically, it can be extended to hundreds of wavelengths) while only relying on the focal plane image. In addition, because the method combines the identification and correction of the co-phase error, the absolute position accuracy of the adjustment platform used for the adjustment of the submirror is reduced, and the resolution requirement is only retained. This method can directly correct the segmented mirror piston co-phase error without iteration after training. The simulation results show that the well-trained deep reinforcement learning algorithm can automatically correct the piston co-phase errors of each submirror in the six submirrors’ segmented system to less than λ/20 (RMS) in the 10-wavelength range by relying only on a simple actor strategy network and focal plane images. At the same time, we also prove the effectiveness of multi-agent reinforcement learning in this method, so that this method can be applied to segmented mirror systems with more submirrors in the follow-up research work. In the future, we will improve the network structure and reward function to improve the efficiency of the method presented.

## Data Availability

Data are unavailable due to privacy.

## References

[1] Devaney N., Kenny F., Goncharov A.V., Goy M., Reinlein C. (2018). Development of a prototype active optics system for future space telescopes. Appl. Opt..

[2] Yaitskova N., Dohlen K., Dierickx P. (2003). Analytical study of diffraction effects in extremely large segmented telescopes. J. Opt. Soc. Am. A.

[3] Koch J.A., Presta R.W., Sacks R.A., Zacharias R.A., Bliss E.S., Dailey M.J., Feldman M., Grey A.A., Holdener F.R., Salmon J.T. (2000). Experimental comparison of a Shack–Hartmann sensor and a phase-shifting interferometer for large-optics metrology applications. Appl. Opt..

[4] Roddier F. (1988). Curvature sensing and compensation: A new concept in adaptive optics. Appl. Opt..

[5] Lilley S., Wizinowich P., Mawet D., Chun M., Cond C., Wallace J., Jovanovic N., Delorme J.-R., Jacobson S., Taheri M. Near-Infrared Pyramid Wavefront Sensor for Keck Adaptive Optics: Opto-Mechanical Design. Proceedings of the SPIE Astronomical Telescopes + Instrumentation.

[6] Gary C., Mitchell T., Nasrat R. Phasing the segments of the Keck and Thirty Meter Telescopes via the narrowband phasing algorithm: Chromatic effects. Proceedings of the SPIE Astronomical Telescopes + Instrumentation.

[7] Li D., Xu S., Qi X., Wang D., Cao X. (2018). Variable step size adaptive cuckoo search optimization algorithm for phase diversity. Appl. Opt..

[8] Li D., Xu S., Wang D., Yan D. (2019). Large-scale piston error detection technology for segmented optical mirrors via convolutional neural networks. Opt. Lett..

[9] Li D., Xu S., Wang D., Yan D. (2019). Phase diversity algorithm with high noise robust based on deep denoising convolutional neural network. Opt. Express.

[10] Chanan G., Ohara C., Troy M. (2000). Phasing the mirror segments of the Keck telescopes II: The narrow-band phasing algorithm. Appl. Opt..

[11] Robert A.G. (1982). Phase Retrieval and Diversity In Adaptive Optics. Opt. Eng..

[12] Durech E., Newberry W., Franke J., Sarunic M.V. (2021). Wavefront sensor-less adaptive optics using deep reinforcement learning. Biomed. Opt. Express.

[13] Martin Levine B., Kaplun M., Ribak E.N. (2022). Asymmetric sparse telescope. Opt. Contin..

[14] Sutton R., Barto A. (1998). Reinforcement Learning: An Introduction.

[15] Guerra D., Trujillo-Sevilla J., Rodríguez-Ramos J. (2020). Towards Piston Fine Tuning of Segmented Mirrors through Reinforcement Learning. Appl. Sci..

[16] Silver D., Huang A., Maddison C.J., Guez A., Sifre L., van den Driessche G., Schrittwieser J., Antonoglou I., Panneershelvam V., Lanctot M. (2016). Mastering the game of Go with deep neural networks and tree search. Nature.

[17] Schulman J., Wolski F., Dhariwal P., Radford A., Klimov O. (2017). Proximal Policy Optimization Algorithms. arXiv.

[18] Radford A., Wu J., Child R., Luan D., Amodei D., Sutskever I. (2019). Language Models are Unsupervised Multitask Learners. OpenAI blog.

[19] Schulman J., Moritz P., Levine S., Jordan M., Abbeel P. (2015). High-Dimensional Continuous Control Using Generalized Advantage Estimation. Comput. Sci..

[20] Chanan G., Troy M., Dekens F., Michaels S., Nelson J., Mast T., Kirkman D. (1998). Phasing the mirror segments of the Keck telescopes: The broadbandphasing algorithm. Appl. Opt..

[21] Ma X., Xie Z., Ma H., Xu Y., Ren G., Liu Y. (2019). Piston sensing of sparse aperture systems with a single broadband image via deep learning. Opt. Express.

[22] Tang J., Ren Z., Wu X., Di J., Liu G., Zhao J. (2021). Object-independent tilt detection for optical sparse aperture system with large-scale piston error via deep convolution neural network. Opt. Express.

[23] Guerra-Ramos D., Díaz-García L., Trujillo-Sevilla J., Rodríguez-Ramos J.M. (2018). Piston alignment of segmented optical mirrors via convolutional neural networks. Opt. Lett..

[24] Jiang J., Zhao W. (2016). Phasing piston error in segmented telescopes. Opt. Express.

[25] Zhao W., Zeng Q. (2017). Simultaneous multi-piston measurement method in segmented telescopes. Opt. Express.

[26] Zhang L., Zhao W., Zhao Y., Liu J., Chu C. (2023). Non-redundant MTF distribution method for sensing multi-piston simultaneously in segmented telescopes. Opt. Commun..

[27] Li D., Wang D., Li J. (2024). Large Range of a High-Precision, Independent, Sub-Mirror Three-Dimensional Co-Phase Error Sensing and Correction Method via a Mask and Population Algorithm. Sensors.

[28] Ma X., Xie Z., Ma H., Xu Y., He D., Ren G. (2020). Piston sensing for sparse aperture systems with broadband extended objects via a single convolutional neural network. Opt. Lasers Eng..

[29] Chanan G., Pintó A. (2004). Efficient method for the reduction of large piston errors in segmented-mirror telescopes. Appl. Opt..

[30] Littman M.L., Cohen W.W., Hirsh H. (1994). Markov games as a framework for multi-agent reinforcement learning. Machine Learning Proceedings 1994.

